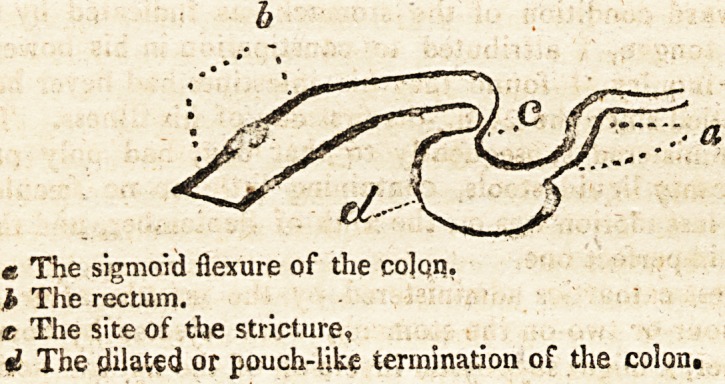# Medical Miscellanies

**Published:** 1818-03

**Authors:** 


### PART IV.

### MEDICAL MISCELLANIES.

### THE PORTE FEUILLE, No. XII.

OR,

DEPOT FOR DETACHED FACTS AND INTERESTING PHÆNOMENA, OCCURRING IN THE PRACTICE OF THE FRIENDS OF THE MEDICO-CHIRURGICAL JOURNAL.

"——— Trahit quodcunque potest
Atque addit acervo ———"

### *Case of unusual Tumour in the Os Frontis and Sternum.*

J. L. a negro, 24 years of age, and in the general enjoyment of good health, had, for several days, been ill of diarrhœa. On that complaint leaving him, a small hard indolent tumour, about the size of a hazel-nut, appeared on the left side of the forehead, over the frontal sinus, attended with exquisite pain in the part, shooting through the whole head.

About a fortnight afterward, a similar tumour appeared on the middle of the Sternum. Poultices were applied to both, but they suppurated very slowly. In the course of a few weeks, however, they did suppurate,—were opened, and an unhealthy glairy pus flowed out, in considerable quantity. The incisions never healed: a little red fungus constantly sprang up betwcen their edges, though ever so often reduced by caustic. On introducing a probe, I found the frontal bone carious, and scabrous as a honeycomb.

This man had been cured fourteen months before, of a venereal complaint, after being several weeks under mercury. But during the time he was under my care, he had no syphilitic symptoms about him whatever, unless the tumours should be regarded as such. The treatment employed consisted in the exhibition of two grains of calomel morning and evening, with ℥ij of strong bark-mixture thrice a-day. The sores were dressed simply with basilicon ointment.

Though these remedies were persevered in for a good many weeks, and his gums became affected, no exfoliation took place, nor did the sores heal; yet they did not ulcerate, but continued in the state of small indolent abscesses. On probing the abscess over the centre of the sternum, the instrument passed in its whole length without resistance, apparently through a penetrating hole in the bone: the margin of this hole felt rough and cellulated.

I regret that I cannot give the termination of this case, as the patient passed from under my care while affairs were in the situation above described. When I last saw him, his general health was not materially affected.

What was the nature or character of these tumours?—I was much at a loss upon this point, at the time; but from subsequent reflection I am inclined to consider them the *spina ventosa* of the Arabians; though, I believe, the latter complaint attacks almost exclusively the ends of the long bones.—I would esteem it a favour if any of the Readers of the Medico-Chirurgical Journal would state their experience of similar cases. R.

---

### *Case of singular Stricture in the Sigmoid Flexure of the Colon.*

J. C. ætat. 30, was taken ill of West-India Fever, of the 2d grade, on the 24th of September. Exciting cause was exposure for nearly a whole day to the beating rays of a tropical sun. The symptoms being pretty urgent, he was bled, the first day, to forty ounces, was freely purged, and had the cold bath very often administered. On the second and third days, a blister was applied to his head, and another to the nape of the neck; his feet and legs occasionally immersed in warm water, and he took the effervescing mixture with spirit. æther. nitros.; and oft repeated doses of calomel.

On the 27th (being the fourth day of the disease) in the morning, I found a complete remission of all the symptoms. Pulse 80. Heat natural, and intellect clear. All kind of medicine was now wholly omitted, because I had found from uniform and pretty large experience, that, after a remission fairly took place, the patient almost always did better when let quite alone, and when no precaution was taken save keeping his bowels open, and at the same time restricting him rigorously to low diet, than when his weak stomach was clogged with bark, and his impaired powers of digestion excited by wine or overloaded by aliment.

In this case, however, the unhappy patient never recovered any degree of strength or appetite, although his pulse was natural, and his mind rational to the last. On the contrary, his tongue continued covered with a thick yellow layer, which shewed no sign of *breaking up*, as it usually does at the end of a febrile paroxysm; but remained immoveable, except towards the edges.

This untoward condition of the stomach, as indicated by the state of the tongue, I attributed to constipation in his bowels; for, on strict inquiry, I found that his intestines had never been properly emptied since the 24th, the first day of his illness. The laxatives administered subsequently to that day, had only produced thin scanty liquid stools, containing little or no fæculent matter. His last motion was on the 27th of September, and then it was a very imperfect one.

The strongest cathartics administered by the mouth, after remaining an hour or two on the stomach, were rejected by vomiting,—or rather, I might say, by an inversion of the natural action of the upper portion of the alimentary canal; for the vomiting seemed scarcely to excite an effort, and was attended with no straining whatever. Purgative enemata were also successless; they could never be retained in any quantity, or long; and came away unchanged. The patient lingered in this distressing state until the 2d of October, (the eighth day from his first attack) and then died without a struggle. His eyes and his skin in several parts previously became quite yellow.

*Sectio Cadaveris.* On opening the abdomen, the first thing that struck me was the state of the liver; this viscus was very considerably enlarged, and had a light grey colour.

The stomach was much distended with liquid ingesta and with air. The intestines were full of fæces and flatus. The ilium, about its middle, was so contracted in its calibre that the little finger could not be passed along without the use of force. No part of the alimentary canal exhibited any traces of recent inflammation.

The arch of the colon was very small throughout, and constricted in several places. Where its sigmoid flexure ends in the rectum, the most remarkable contraction existed. The lower portion of the flexure was dilated into a pouch or bag, and the continuous commencement of the rectum was so very small as barely to admit the passage of the little finger. It had the precise appearance of the colon's ending in a *cul-de-sac;* and, although the canal was not absolutely impervious, still, I have no doubt, that this singular stricture amounted to a complete obstruction during the last days of his life, and was the main cause of bringing the latter to a premature termination.

These morbid changes, as far as I could judge, did not seem the result of recent disease, for there were no signs of inflammation either acute or chronic,—either of short or long standing. Indeed it struck me, that they were the work of years!

*Query.* How far might these various and unusual strictures be owing to his trade as a house-painter? To this business he had been brought up, and had been pretty constantly employed in it from his youth.

By the time these morbid appearances were discovered, I had no means of ascertaining whether this man had ever been subject to intestinal spasms, or to symptoms of *colica pictonum.*

The following is a rude representation of the parts;

*a* The sigmoid flexure of the colon.
*b* The rectum.
*c* The site of the stricture,
*d* The dilated or pouch-like termination of the colon.

---

### MISCELLANIES.

Quicquid agunt Homines.

---

### *Refutation of Sir* William Adams's *alleged Charges against* Mr. Stevenson.

---

*To the Editors of the Medico-Chirurgical Journal & Review.*

Gentlemen,

I cannot but consider it just cause for self-congratulation, that Sir William Adams, whose name I have never introduced into any of my Publications, and with whom I have not the slightest personal acquaintance, but who seems, from the list of highly respectable medical practitioners he has made the objects of his animadversions, to be a man,

"Jealous in honours, sudden and quick in quarrel"; after scrutinizing my conduct and successful career with eagle-eye, is at length able only to adduce against me the truly grievous charge of having, *more than seven years since,* anticipated him in the *alteration* and *use* of a Surgical Instrument! The accusation is, indeed, so frivolous and unimportant, that, had I not been called upon in the Review of Sir William's recent production, which appeared in the last number of 'The London Medical and Physical Journal,' in which his *pretensions to originality* are duly appreciated and exposed; and deemed it possible, that my silence might be disadvantageously interpreted by some of the *numerous extra*-professional persons, to whom the Letter and Supplement, containing "the ground and front of my offending," have been *gratuitously* distributed by the author, I should not have sacrificed that time in noticing it, which can be so much more agreeably and beneficially employed. But this allegation, insignificant as it is, when stript of the imposing effect of dates, garbled quotations, and gratuitous assertions, is founded upon a presumption as improbable and extravagant as, from the sequel it will be found, untrue. For can it be conceived for a moment, by the most credulous, that an apothecary, whatever his talents and inclination, after *once only* witnessing an operation for cataract, *which he had never before seen*, could be capable of impressing *my* mind with such a distinct idea of the several steps of that process, and of the particular form and mechanism of the instrument, &c. as to enable *me* accurately to detail the one, and to describe and delineate the other? Conclusive as this view of the question may appear, my exculpation does not rest on merely presumptive evidence, or speculative and abstract reasoning. I have had an interview with the apothecary (whose address I fortunately procured through the medium of a patient to whom the aforesaid pamphlets were sent with the *name*, which is carefully *suppressed* in the *text*, written by the pen of Sir William himself, at the foot of the page), who is made, without his knowledge, to act so prominent a part in this singular Drama, and upon whose *presumed communication* the whole weight of Sir William's arguments, and *my implied* guilt principally depend. That worthy and very respectable gentleman authorizes me to state, and to refer any one to him for further information, "that though, as a casual and disinterested visitor on the occasion alluded to, he had no motive for retaining in his recollection the particulars of a transaction which took place so many years since, and with which he had not the smallest concern, he has no hesitation in declaring his conviction, as he could not, from what he might observe, or from his own previous knowledge of the operation, hope to add any thing to my intimate acquaintance with the subject, that the insinuation, which is so pointedly directed against me, is not only purely imaginary, but utterly destitute of foundation.!" This declaration, which I can most conscientiously confirm, at once acquits me of having obtained, thro' that channel, the information relating to the *construction* and *method of using my needle*, so illiberally imputed to me by Sir William Adams! I may likewise add, as a further proof, if any were still wanting, of the groundlessness of the aforesaid charge, that "the curious coincidence" on which Sir William lays such great stress, between the period when he performed the operation in Portman Square, viz. in March, 1811, and the time at which the *alteration* in the form of Mr. Saunders's needle, is stated to have been directed by me, applies by virtue only of the *omission*, on the part of Sir William, of *two small*, but, on this occasion, *not unimportant words*, which are inserted, page 77 of the first edition of my "Practical Treatise on Cataract," from which passage his quotation is derived. As these luckless and discarded words, "*upwards of*," were intended to convey an indefinite meaning, not imagining, at the time they were written, that extreme accuracy on the score of dates was indispensable, the following note from my instrument maker, Mr. Ewing, (who has prepared, and will be ready to exhibit comparative specimens of Sir William's, and my needle, and speculum; and also the various surgical instruments altered and invented by me, for the purpose of facilitating different operations on the *eye* and *ear*) will show, not only that *my needle existed*, and of its *present shape* too, *many months prior* to the *earliest* period assigned for its construction by Sir William Adams, upon the most vague surmises, but that it differs likewise in one important point of its mechanism, from that of Sir William Adams. The cases which suggested that improvement, and which occurred so long since as the year 1809, together with the advantages resulting from the subsequent alteration of the needle, are explained at large, (with several particulars relative to the nature of the disease, *black* cataract, the mode of conducting the operations, after-treatment, &c. not even adverted to by Sir William) at page 103 et seq. and 147 of the *second* edition of my "Practical Treatise on Cataract."*

"Drury Lane, Feb. 14, 1818.

"Sir,

"I am ready to assert, that your needle was made in the year 1810, and that it may be distinguished by being *round* at the part where Sir William Adams's is *flattened*.

"I remain, Sir,

"Your most obedient Servant,

"William Ewing."

"*To John Stevenson, Esq.*"

With regard to my *Speculum*, which has been always represented as *an alteration only* of Pellier's, little need be said; since I have long discontinued the use of all *mechanical* assistance for steadying the eye. I cannot, however, forbear to observe, that, had Sir William taken the trouble only to insert *my* description of it, as given, page 129 of the second edition of my "Treatise," and the faithful back and front view prefixed to that publication, instead of presenting us with a *simple wooden outline sketch*, which conveys just as exact a notion of its real form and character, as a circle would the figures and superscription on the face of a medal, I should have escaped all suspicion of plagiarism.

What has been advanced is, I presume, quite sufficient to refute the unwarrantable charge brought against me by Sir William

* This work, (published by Longman and Co. Pater-noster Row, and Harwood, Great Russel Street), contains likewise a well-engraved plate of my needle, and a copy of Mr. Saunders's, to shew the difference of their respective construction; also of my speculum, and iris or capsule knife, and the *only authentic* document left on record, and sent to me in a letter, by my worthy friend, the late ingenious Mr. Saunders, relative to *his* modes of operating in the different species of cataract.

Adams, of having availed myself, "not only of *his* operation for soft cataract, but likewise of the instruments employed by him in its execution."

In short, had Sir William Adams, before venturing to make this equally unprovoked and unjustifiable attack, done me the justice to ascertain by application to the parties above alluded to, the truth or falsehood of his statements, I should have been spared the irksome task of offering this public appeal, in vindication of those claims which are proved to have existed long before any publication of Sir William Adams had issued from the press; the earliest of which (on Diseases of the Eye) did not appear until the middle of August, 1812!

Sed magna est veritas, et prævalebit.

I am, &c.

JOHN STEVENSON.

*Great Russell Street,*
*Feb.* 16, 1816.

---

*Committee Room, Crown and Anchor Tavern, February* 4, 1818. At a meeting of the Committee of the Associated Apothecaries and Surgeon Apothecaries of England and Wales, Mr. Parkinson in the Chair, The Committee having endeavoured to ascertain the most appropriate means of securing the objects of this Association, and having resolved on the measures at present to be adopted, further agreed unanimously, to the following resolutions. 1st. That it is exceedingly gratifying to observe, that the public health and the respectability of the profession are much protected by the act of Parliament, originating with the former Committee of this Association, and procured through the exertions of the Society of Apothecaries. 2d. That the public advertisements of the Society of Apothecaries, declaring their determination to prosecute any person, practising as an apothecary, in violation of the said act, will, it is presumed, have considerable influence in preventing the intrusion of unqualified pretenders into the practice of medicine. 3d. This Committee regards with great satisfaction, the liberal manner in which public lectures have been instituted by the Royal College of Surgeons, and by the Society of Apothecaries; convinced that the public welfare, as well as of the best interests of this Association, must be promoted by the extension of professional knowledge. 4th. That the preceding resolutions be transmitted to the several Medical Journals.

---

*Drs. Gall and Spurzheim's System having been rather roughly handled by the Edinburgh Review, in their article on this subject, Dr. S. has written a Reply, in vindication of his Doctrine, from which we insert the following Extract.*

"In order to show to the faithful readers, the inexact report of the Edinburgh Reviewer, I shall confront only a few passages of my book with the statements suggested by him to the public. First, as to the object of our investigations, he found it suitable to state, that the physiognomical part of our inquiries "teaches how to find out, by the shape of the head, whether a man loves his children, or kills them; whether he steals, or is very benevolent." P. 250.—We, however, continually maintain, that we never can speak of the actions of man; and after having mentioned the title, Physiognomical System, I begin the introduction of my book, "This system is commonly considered as one, according to which, it is possible to discover the particular actions of individuals; it is treated as an art of prognostication. Such, however, is not the aim of our inquiries; we never treat of determinate actions; we consider only the faculties man is endowed with, the organic parts by means of which these faculties are manifested, and the general indications which they present."

"Moreover, the Reviewer tells his readers, that "Gall and Spurzheim, in fact, in affirming that the vigour of intellect is always proportional to the size of the head, seem to have been desirous of trying how far their effrontery might be carried. If they succeed in convincing a single individual of common parts and observation, that this assertion is truth, they will find little difficulty, we apprehend, in persuading mankind in general, that they hear by their eyes and see by their ears." P. 247. The contrary, however, of that assertion is maintained all along my work. I shall extract only a few passages, the pages of which refer to the 2d Edit. which the Reviewer quotes. Pages 190 and 191, I have professedly examined that proposition; and conclude, "It is not, however, possible, even in individuals of the same kind, to measure their faculties according to the absolute size of their brain. Hence, it is necessary to look for other means of determining the degree of the faculties of the mind." Pages 215 and 216, I have said, "In order to judge exactly of our proceeding, it must be considered, that we do not endeavour to determine every degree of activity of any cerebral part, but only the nature of its functions, and to this end its size is sufficient." "I admit even the possibility, that, in the same individual, the internal constitution of the different parts of the brain may vary, in the same way as the optic nerve may be more irritable than the auditory or olfactory." The Reviewer could also read, p. 526, "I have often repeated, that, in speaking of the actions of man, it is not sufficient to consider the size of the organs of the respective faculties, but that the internal organic constitution of the cerebral parts, the exercise of their faculties, and their mutual influence, contribute also to their different degrees of activity."

"Now, I shall not be so illiberal as the Reviewer, and accuse the "conscientious" man of "*wilful misrepresentations;*" but conceive his having written the article without previous perusal of the book. I shall only add the inaccurate report of our manner of proceeding. "Can it be possible," says the candid examiner, "that the great Drs. Gall and Spurzheim have not observed, in the course of their multiferous inquiries into Nature, that phænomena may coincide, without being related to each other, as cause and effect? Were it established, that all great mathematicians had black eyes, and all poets blue ones, would any sensible man, from this alone, think of ascribing the mathematical talent in the one case, or the poetical genius in the other, to the col·ur of the iris?"

"Had the learned Reviewer studied Chap. I. of Part III. of my book, he would have seen, that we are aware of the difference between coincidence and the relation of cause and effect to each other, and never lose sight of it; that we are not satisfied with single observations, but prove our assertions in the same way as any other physical truth; and that, being convinced that nature is constant in its laws, we reject any of our opinions as soon as we find one exception. If, however, an observer could show, that only mathematicians have black eyes, and only poets blue ones; that every one who has black eyes, and no one but those, have mathematical talents; or that every one with blue eyes, and only those, are born poets: if he could repeat his observations in various countries; if he could compare the same talents through a series of animals, without finding an exception; if he could support his observations by other means which I have detailed in my book, he might establish a Physiognomical Sign, and provoke his opponents to show the contrary. So we do. Hence, he who will refute our assertions, founded on facts, ought not to be satisfied with speaking merely of absurd theories, incredible and disgraceful nonsense, trash and despicable trumpery; he ought not to write a libel of forty-one pages, full of personal invectives, calumnies, and abuses; he ought not to speak of motives and consequences of these investigations; ought not to invent suggestions, and combat them; ought not to have recourse to ridicule and sophistry, but show plain facts. If, for instance, we speak of a sign of self-esteem, let us see that a man, the most prominent feature of whose character is composed of self-conceit, does not offer the sign on his head. One fact to the contrary, well observed, will be to me more decisive than all metaphysical reasoning of the schools, or a thousand captious or ludicrous opinions.

"These few remarks, which I could easily multiply, may be sufficient to prove, how punctually the Edinburgh Reviewer has shown "the conscientious discharge of his duty on this occasion." p. 229.

---

DEATHS.—In Stone Street, Maidstone, aged 69, Thomas Day, M. D. In Finsbury Square, Dr. Da Costa. In the 41st year of his age, James Aleyne, Hendy, M. D. Suddenly, at the house of one of his patients, Sir Richard Croft, Baronet.

## Figures and Tables

**Figure f1:**